# Exploring the Ethics of Implementation of Epigenomics Technologies in Cancer Screening: A Focus Group Study

**DOI:** 10.1177/25168657211063618

**Published:** 2021-12-09

**Authors:** Eline M Bunnik, Ineke LLE Bolt

**Affiliations:** Department of Medical Ethics, Philosophy and History of Medicine, Erasmus MC, University Medical Centre Rotterdam, Rotterdam, The Netherlands

**Keywords:** Cancer or oncology, medical screening, focus group research, medical ethics, epigenomic technology, responsible innovation

## Abstract

New epigenomics technologies are being developed and used for the detection and prediction of various types of cancer. By allowing for timely intervention or preventive measures, epigenomics technologies show promise for public health, notably in population screening. In order to assess whether implementation of epigenomics technologies in population screening may be morally acceptable, it is important to understand – in an early stage of development – ethical and societal issues that may arise. We held 3 focus groups with experts in science and technology studies (STS) (n = 13) in the Netherlands, on 3 potential future applications of epigenomic technologies in screening programmes of increasing scope: cervical cancer, female cancers and ‘global’ cancer. On the basis of these discussions, this paper identifies ethical issues pertinent to epigenomics-based population screening, such as risk communication, trust and public acceptance; personal responsibility, stigmatisation and societal pressure, and data protection and data governance. It also points out how features of epigenomics (eg, modifiability) and changing concepts (eg, of cancer) may challenge the existing evaluative framework for screening programmes. This paper aims to anticipate and prepare for future ethical challenges when epigenomics technologies can be tested and introduced in public health settings.

## Introduction

Epigenomics is the study of epigenetic mechanisms across the genome. One of the best-studied common epigenetic mechanisms is DNA methylation, the addition of methyl groups to specific locations of the DNA molecule in response to environmental stimuli, including exposures, diet and lifestyle. DNA methylation affects transcription and, therewith, gene expression. It may inactivate or ‘silence’ genes. When epigenetic changes switch off tumour suppressor genes, the risk of cancer increases. The pattern of DNA methylation in human cells may thus serve as a set of biomarkers for cancer. Around the world, researchers are learning about the relationships between epigenetic changes and various types and stages of cancer, and developing and validating DNA methylation tests for the diagnosis or prediction of cancer.^1^ At the same time, epigenome-wide technologies are being developed that allow researchers to determine the DNA methylation pattern of the entire genome (the ‘epigenome’) more and more reliably, rapidly and affordably.^2,3^ Due to these twin scientific and technological advances, epigenomic tests might become suitable not only in clinical settings, to improve the diagnosis and staging of cancer in patients, but also in public health settings, to predict cancer risk in healthy individuals. With this, implementation of epigenomic tests in population-based cancer screening comes into view. For instance, there is a growing body of evidence suggesting that screening tests for cervical cancer based on DNA methylation patterns may be of higher sensitivity and/or specificity than current tests based on detection of infection with specific strains of human papillomavirus.^4-7^ In cervical cancer screening programmes, which have been offered in many European and other high-resource health care systems since the 1960s,^8^ epigenomics-based tests may come to complement or replace current screening modalities.

Implementation of epigenomic technologies in cancer screening programmes raises ethical issues. Epigenomics has 3 characteristics that set it somewhat apart from other biomedical data. Firstly, as epigenetic changes may result from environmental stimuli, epigenomic data may convey information about environmental exposures, living conditions, past health-related behaviours, such as diet or (mis)use of tobacco, alcohol and other substances and childhood trauma. This information may be of particularly sensitive nature, especially when epigenomic tests indicate that disease has been caused by wilful acts, resulting in feelings of guilt,^9^ regret or shame. Secondly, some epigenetic changes are heritable and can be passed on to the next generation – and generations thereafter. Through epigenetic mechanisms, life circumstances, lifestyle and life choices may leave traces not only on our own bodies, but also on those of our children and grandchildren and so on. The epigenome has been referred to as our ‘bioarchive’.^10^ This possibility has sparked discussions on individual and social transgenerational responsibility,^11,12^ although confirmation of findings suggestive of intergenerational transmission of epigenetic information in humans is still pending.^13^ Thirdly, unlike genetic mutations, epigenetic changes can be influenced by lifestyle factors, and change over time. As epigenetic changes are modifiable and reversible, testing for epigenetic risk – in principle – yields actionable results. By leading healthier lives, individuals may restore high-risk epigenetic markers to normal and lower their cancer risk. In theory, its modifiability renders epigenomic cancer risk screening suitable for maintaining and improving the health of populations.^14^ This characteristic has also set off a debate on personal responsibility, blame or moral sanctions^15-18^ and the risk of stigmatisation or discrimination.^19^

It should be noted that there are various obstacles that may hinder or even halt the development and application of epigenomic technologies in public health settings. For instance, as the epigenome is unstable, adequate assessment and monitoring of cancer risk may require repeat or frequent evaluation of the epigenome.^14^ Also, patterns of epigenetic changes differ across cell types within one individual, such that the epigenetic changes within tumour cells that are typical for malignancy, may not occur in blood, saliva or other easily accessible tissue. However, epigenetic changes detected in cervical cells, for instance, are believed to be reliable indicators for tumours in the breast, ovaria and uterus,^20^ and are relatively accessible through cervical swabs. Several epigenomics-based tests are already used in clinical laboratories, for instance for the early detection of colorectal cancer and for the diagnosis of neurodevelopmental disorders of genomic imprinting.^10^

A European research consortium is currently developing multi-omics based testing, including epigenomic testing, to be used within a risk-stratified approach to screening for female cancers – breast, cervical, endometrial and ovarian cancer.^21^ The proposed approach implies that the eligibility of individual women for the screening programme, as well as the frequency and screening modalities are conditional upon and tailored to individuals’ cancer risks, as determined by an – in part epigenomic – risk prediction screening test.^21^ Consequently, individuals with a low risk of cancer can be screened less frequently than individuals with a high risk of cancer, and over-screening and over-diagnosis could be reduced. When taking part in such a screening programme, women would receive individual risk prediction information on 4 female cancers, which may be difficult to interpret.^22^ Also, the test may lead to incidental findings, and epigenomic information may reveal sensitive information about past exposures, life and lifestyle.^23^

In the near future, epigenomic technologies might take the place of conventional testing modalities within existing screening programmes, such as cervical screening programmes, based on better test performance.^5^ Alternatively, they may be coupled with other omic technologies (eg, polygenic risk scores) to improve risk stratification (based on eg, age or lifestyle factors) within screening programmes.^24^ In combination with genetic/genomic and other omic technologies, epigenomic technologies might be used to change the nature of existing cancer screening programmes, as in the screening programme for 4 female cancers, allowing for the simultaneous assessment of multiple cancer risks in one test. According to some scientists, it might become possible to use epigenomics technologies for what is referred to as ‘global cancer screening’ based on a blood draw and the analysis of DNA methylation patterns in circulating cell-free DNA in blood (a ‘liquid biopsy’).^25^ Some applications are already being developed: CancerSEEK, for example, uses multi-analyte tests to detect 8 early cancers.^26^ Theoretically, in the future, an annual or biennial blood draw would suffice to screen for all cancer types and/or to monitor the effects of risk-reducing lifestyle interventions on the risk for all cancer types.

In this paper, we set out to explore ethical issues associated with the use of epigenomic technologies in cancer screening. We report the results of an exploratory focus group study among scholars with expertise in the evaluation of emerging (bio)medical technologies, aimed at the identification of ethical issues associated with 3 potential future applications of epigenomics technology in population-based cancer screening. In parallel with presenting the results of the focus group study, we critically discuss key issues raised by the experts, identify concerns and challenges for implementation of epigenomic technologies in screening programmes, and suggest approaches to address these concerns and challenges.

## Methods

Focus groups are carefully planned series of group discussions in which respondents’ views are discussed in an informal, permissive atmosphere, using the dynamics of the group ‘to prompt fuller and deeper discussion and the triggering of new ideas’.^27^ As a qualitative method, focus groups are especially suitable for the exploratory investigation of respondents’ views on new or emerging topics,^28^ such as epigenomics technology. The study was designed and conducted in accordance with the COREQ guidelines.^29^

### Ethics

In the Netherlands, focus group studies with experts are not subject to the Dutch Medical Research Involving Human Subjects Act. Ethics approval therefore was not necessary or possible. All respondents provided informed consent verbally for participation in the study, for audio- and/or video-recording of the discussion and for publication of the results.

### Setting and sampling

Taking a purposive sampling approach, the research team contacted the ethics and/or philosophy departments at Dutch universities in the Netherlands by e-mail asking if we could organise focus group discussions with researchers with expertise in the ethical and societal evaluation of emerging (bio)medical technologies. Focus groups were held at work locations of universities or online – due to national lockdown measures during the COVID-crisis – between November 2019 and March 2020. Focus groups lasted approximately 90 minutes. We prepared for the online focus group using the reported experiences of other research groups with online focus groups, some of which involved experts working in biomedicine.^30-34^ Holding the focus group at an online platform, in our experience, was feasible. Any dissimilarities we observed with the dynamics of face-to-face focus group discussions were unproblematic for the conduct of the study. As all 3 focus groups included respondents who did not speak Dutch, discussions were held in English.

Respondents included 8 women and 5 men (n = 13) in total. Five respondents were either associate professor or full professor, while 2 were assistant professor, 5 were postdoctoral researchers and 1 was a PhD-student. Respondents were trained in philosophy of science, philosophy of technology, bioethics, ethics of technology and/or science and technology studies (STS).

### Data collection

In each focus group, we discussed 3 potential future applications of new epigenomic technologies in public health settings:

1. Epigenomics-based cervical screening, based on a cervical smear;
2. Multi-omics-based screening for 4 women-specific cancers, similarly based on a cervical smear, and;
3. Annual global cancer screening, based on a liquid biopsy.

We asked respondents to discuss the opportunities and challenges that arise in each of these areas of application from an ethical point of view. The group discussions were audio-recorded. The recordings were transcribed verbatim, and anonymised.

### Analysis

The first 2 transcripts were analysed thematically – inductively, by assigning and reassigning codes, and grouping codes together under themes – by 2 researchers (EB and IB) independently, using a constant comparative approach.^35^ Discrepancies in codes and themes were discussed and resolved, and the code book was finalised. Analysis of the third transcript (by EB) did not give rise to the creation of new codes or themes. Theoretical saturation was reached.

## Results

Focus group participants recognised the potential of epigenomic technologies to enhance cancer screening programmes by contributing to test performance (eg, sensitivity and specificity) of screening modalities and to risk stratification. They noted that epigenomics-based screening could help to avert over-diagnosis and over-treatment, and to improve the balance of risks and benefits of cancer screening by tailoring preventive health measures to individual risks and allowing screening participants to monitor the effects of preventive measures over time. At the same time, respondents were primarily concerned about 4 sets of issues, namely epigenomic risk communication, informed consent, and trust; responsibility, stigmatisation and blame; epigenomic data protection and data governance and; the adequacy of the existing ethical framework for population screening to accommodate potential changes brought on by the introduction of epigenomic technologies in public health settings.

### Theme 1: Risk communication, informed consent and trust

Respondents were concerned that the level of knowledge and familiarity of epigenetics and epigenomics among the general public may be limited, and that the biological and technical complexity of epigenetic mechanisms would hinder adequate information provision and informed consent for cancer screening. It takes a high level of scientific literacy to understand how epigenomics-based screening works. Respondents wondered whether face-to-face discussions with general practitioners or other primary care health professionals would be required, and whether these professionals would be able to explain what epigenomic screening entails and what the results might mean. Informed consent was deemed important; before starting on the trajectory of screening, people need to understand ‘the whole chain’ (focus group B, application 2) of events or decisions they may be confronted with based on the possible outcomes of screening. Respondents suggested that those working in population screening start thinking about ‘a kind of dashboard interface design’ (focus group A, application 1) to provide information about epigenomics-based screening effectively and efficiently.

Respondents noted that whereas most population screening has traditionally aimed at the early detection of disease, epigenomics-based screening allows for risk prediction in healthy individuals for diseases that have not (yet) manifested at all. Epigenetic risk prediction however was not seen as unique and was compared to genetic and other biomarker-based testing and screening in health care. Respondents were concerned that in general, participants may have difficulties understanding predictive information about disease risks, and that misunderstanding of risk information would lead to adverse psychological or health effects.

In relation to applications 2 and 3, these concerns were exacerbated, as multi-cancer screening programmes entail that healthy individuals are confronted with risk predictions on various types of cancer. In an epigenomics-based women-specific screening programme, for instance, a woman might learn that she has a high risk of breast cancer, a low risk of cervical cancer and an average risk of ovarian and endometrial cancer. This woman would be advised to attend breast cancer screening more frequently, and that she needs no further screening for cervical cancer. Respondents expected especially the latter to be problematic:

*R1: It will be very hard, once you have offered [screening] to people, to start [taking it back]. . .*

*R2: [But these are] people who don’t need it..*

*R3: Exactly..*

*R4: But [still they may feel that they] have a right [to screening].*

*R1: I’m not saying it’s impossible, but you might meet some opposition here. [. . .] This is a nice example of how people start experiencing things as moral rights. (Focus group B, application 2)*

Further, it was feared that lack of understanding among the target group of a screening programme might result in distrust and noncompliance or low uptake of screening. It was anticipated that people’s interest in taking part in epigenomics-based cancer screening would depend on the way in which it would be presented to the target group, with a focus on the relevance of screening. The relevance of screening was believed to be conditional upon the actionability of the result. The results of screening should be clear (ie, clinically valid) and people should be provided with clinical follow-up, such as tailored screening programmes or clear risk-reducing lifestyle recommendations.

Finally, it was mentioned in our focus groups that epigenomic screening tests may reveal information about other, unrelated conditions, that is, incidental findings. Some incidentally uncovered information may be especially sensitive, as it may unveil environmental and lifestyle exposures. As this information may be unwanted, it was believed to be important to clarify screening participants’ preferences for the return of incidental findings as part of the informed consent process. Again, respondents noted similarities with genetic and genomic testing and screening.

### Theme 2: Responsibility, stigmatisation and blame

Because of its relation with environmental and lifestyle exposures, it was felt that epigenomic information may have a special impact on individuals and institutions. Based on emerging knowledge of causal relationships between specific environmental and lifestyle exposures and epigenetic changes, cancer risk may become attributable to such exposures. These exposures may or may not have been within the sphere of control of the individual, and may thus lead to self-blame or blaming and liability of third parties, respectively. If one’s (increased risks of) cervical cancer can be attributed to a history of smoking, for instance, individuals may hold themselves responsible for their disease (risk) or may be held responsible by others. If one’s (increased risks of) cervical cancer can be attributed to environmental exposures, the responsibility might lie with third parties. Respondents pointed out that advances in epigenomic science and technology would thus affect the distribution of individual and social responsibilities. Specifically, epigenomic-based cancer screening would open up ‘discourses of blame’:

*R1: But if I get a risk profile, I don’t know who to blame.*

*R2: Or what to blame.*

*R3: And you might either blame yourself, or you might blame your mother, or you might blame whoever. I mean, at any rate, it raises the blame question. (Focus group B, application 2)*

Also, epigenomic data are not stable and repeat assessments may be required to evaluate epigenetic cancer risk over time. The potential for real-time measurements of the biological effects of risk-reducing measures was considered both an advantage and a disadvantage of the use of epigenomic technologies in cancer screening. On the one hand, by making the health effects visible, it could help to motivate screening participants to improve their lifestyles and avoid environmental risk factors. On the other hand, it might imply that adequate cancer risk management will require continuous or frequent tracking of the effects of risk-reducing measures and lifestyle choices through epigenomic monitoring, which could be a significant burden for individuals. Respondents drew attention to broader concerns, noting that the increasing availability of screening and self-testing in general already turns healthy individuals into ‘potential patients’ with various levels of ‘to-be-sickness’ within their bodies, which could be harmful or burdensome for screening participants. Also, individuals may lose the freedom to withdraw from screening; once an individual has started on a trajectory of participation in screening, epigenomic or otherwise, there would be no going back. As one respondent remarked: ‘So you’re signing people up for a lifetime of testing’ (Focus group A, application 1).

In all focus groups, respondents connected these concerns to a conception of epigenomics-based screening programmes as a form of ‘biopolitical control’, in which individual interests and agency must give way to the achievement of societal goals, that is, the health of the population, and individuals are compelled to adopt healthier lifestyles.

*If I were very cynical, I would just see this as a control mechanism. A way to control women and keep us [. . .] healthy foetal containers that then stay healthy to produce offspring. [. . .] Why is it always us being tested? (Focus group C, application 2)*

In all focus groups, the burden on women was discussed. In application 2, the female body was believed to be presented as especially vulnerable, as carrying all sorts of risks. Respondents raised questions with regard to the power hierarchy involved in the frequent or continuous monitoring and measuring of the functioning of the female body. One respondent argues that, for this reason, global screening programmes might be preferable to female screening programmes:

*“The upside [of a global approach] would then be that that risk group would be so broad that you might not feel instantly as scared or at-risk, as [you would] in the more targeted [female] interventions. So again, a bit back to the gender question that women are called into more screenings than men, [women are] already getting a sense of that fact ‘Oh, the female body is somehow a problem’. I mean, [as a woman] you are in danger. So, there are also some upsides to a very global intervention that puts us all, almost as humans, okay, the humans over forty or whatever, into the same category of at-risk”. (Focus group B, application 3)*

### Theme 3: Data protection and data governance

Given the potential sensitivity of epigenomic data, respondents felt that the security of data and samples and the protection of the privacy of screening participants was a crucial condition for responsible implementation of epigenomics technologies in public health settings. Epigenomic information is seen as (potentially) particularly intrusive or infiltrating:

*Now we are constantly measuring the fluctuations of your epigenetic profile for cancer which is affected by where you live, the lifestyle you have, the food you’re eating, potentially everything that you do. It’s really infiltrating. (Focus group A, application 1)*

Respondents anticipated concerns among the general public with regard to epigenomic data protection within screening programmes, and referred to the potential for ‘epigenetic discrimination’ by employers and life or long-term health care insurers.

In relation to application 1, respondents noted that the arising of ethical concerns would be highly dependent on the future design of the epigenomic technology. Would the test necessarily reveal information about the causes of cancer risk, and thus about lifestyle or past exposures, or could it be targeted such that it conveyed information about cancer risk only? In case the test could be targeted, women would merely be subdivided into normal risk and high risk, and high-risk women might require follow-up diagnostic testing or more frequent monitoring, just like in existing screening programmes. There would be one remaining difference, namely that in the epigenomics-based screening programme, women might receive individualised health recommendations aimed at the reduction of epigenetic risk, such as smoking cessation, physical exercise, a healthy diet. However, such health recommendations are likely to be generally applicable to the population, and therefore not specifically distressing. If, on the other hand, the screening programme were to provide women with information about the relation between health and past environmental or lifestyle exposures, it was felt, this would have ‘major implications’ (focus group B, application 1) in relation to privacy. During the development process for epigenomics technology, it was felt, researchers and test developers should take into account the privacy implications of design choices.

Focus group respondents discussed the dynamic nature of epigenomic data and whether the instability of epigenetic markers over time increased or rather decreased the risks to individual privacy. In the future, one’s epigenome may look entirely different. When data collected in a population cancer screening programme are stored for a long duration of time, they may pertain to epigenomic states that may have changed or disappeared in the meantime. Epigenomic data from the past may have lost its predictive ability and may have little to say about one’s present health or risk status. At the same time, the information contained in the data may nonetheless (and unduly) be used by third parties against one’s interests in the present. Thus, the reversibility of the epigenome does not remove the risk to privacy. It does suggest that any clinical (or other) interpretation of epigenomic data should consider time and timing of sampling.

Even if the results of epigenomics-based screening tests would not imply or include information about environment or lifestyle exposures, and epigenomic data are de-identified and stored securely, there is a risk of data leaks or breaches. Respondents were concerned about long-term storage and future possibilities for making inferences based on linkage with data from other (publicly accessible) sources.

### Theme 4: Ethical frameworks for screening

Focus group respondents invoked some of the Wilson and Jungner criteria for the evaluation of screening programmes.^49^ For instance, if an epigenomics-based test requires only a blood draw, it is seen as ‘non-invasive’, and thus more likely to be acceptable to the population. In accordance with the screening criteria, respondents felt that the benefits of screening should outweigh the harms. According to respondents, the benefits of the envisioned epigenomic test included better ability to predict disease (focus group B, application 1), and better insight in the causes of cancer, including the causal interplay between genetic and environmental factors (focus group B, application 3). It was deemed important that the epigenomics-based screening test has adequate sensitivity and specificity.

It was felt that better health outcomes for cancer patients was a broadly shared priority, and that people would be willing to take part in research focussed on research and development of epigenomics-based cancer screening tests:

*But also, to emphasise the positive sides, I guess, unfortunately, everybody knows people who had cancer and have cancer and who are experiencing advantages from the vastly, very quickly developing medical insights. In that sense I think cancer research is probably one of the domains where people are very, very willing to contribute one way or the other. (Focus group A, application 1)*

At the same time, there were concerns with regard to the current lack of specific risk-modulating treatment or preventive options for many diseases. In the absence of opportunities to improve health outcomes, there may be no benefits to outweigh potential harms. Offering screening is ethically acceptable only when screening has the potential to yield actionable outcomes:

*I can imagine that there is something tricky about being more precise and saying: ‘You’re going to get this [disease]. And we think that probably - with the information we have right now - we should also be able to give you more targeted treatment, but we’re not really there yet’. (Focus group A, application 1)*

Some focus group respondents were especially sceptical that the offering of epigenomics-based recommendations to adopt healthier lifestyles would outweigh potential harms. After all, many people are aware of general preventive health recommendations, but fail to act upon them. More precise risk information, as obtained through epigenomic screening, might do little to change that.

*There’s a suggestion that they might also be able to provide lifestyle advice and things like that. And even then, it might be challenging to determine whether that’s sufficient to justify such a huge screening program. (Focus group B, application 3)*

Also, respondents were concerned that because of the dynamic nature of epigenomic information, any result of an epigenomics screening test at a given moment would be difficult to interpret:

*You can’t do away with one screening moment. Say you get an idea about someone’s risk profile in 2019. The risk profile might look very different in 2022, depending on how fast these [epigenetic] processes go. So how - and I’m thinking practically - how is this ever going to be a feasible way of screening, if you screen something that is constantly moving? It’s a moving target that you’re hunting. (Focus group B, application 3)*

Respondents pointed out shifts in the ethical framework for screening that might follow from the introduction of epigenomics technologies in public health settings. The Wilson and Jungner criteria were originally developed for programmes aimed at early detection of one specific disease. The envisioned application of epigenomic technologies in female-cancer programmes, on the other hand, entails a more or less ‘constant tracking of the various levels of possible cancer arising or not in 4 different ways’ (focus group A, application 2). Respondents wondered whether it would be possible to assess multi-cancer or global-cancer screening programmes using the traditional ethical framework for the evaluation of population screening.

## Discussion

This focus group study identifies opportunities for the implementation of epigenomics technologies in cancer screening related to personalised risk prediction, risk stratification and risk modulation, and benefits such as reduction of false positives and over-diagnosis. Also, it points out concerns regarding the ethical implications of the offering of epigenomic risk information in cancer screening programmes.

First, if epigenomic technologies – coupled with other technologies – were to be used in cancer screening for the purposes of risk stratification, the screening programme would need to focus on adequate communication of risk information. Understanding risk information is notoriously difficult for most people. This is a well-known and general problem for population cancer screening, as well as for other forms of medical screening and testing. Ideally, an individual’s taking part in screening should be the result of an autonomous decision, made free from pressure or coercion, and based on adequate – and adequately understood – information. In practice, the latter requirement seems difficult to meet. Participants – particularly those with poor health literacy – may have difficulty understanding the purpose of cancer screening, assessing risk information and weighing the potential benefits and harms of risk-stratified screening.^36^ While the trend towards risk prediction in screening – and in medicine, generally – surely is broader and not unique to epigenomics, epigenomics-based risk-stratified screening may increase the gap between the ideal and the reality of informed, autonomous decision-making by screening participants, because general publics are not familiar with epigenetics. Difficulties surrounding information provision and informed consent may exacerbate further if epigenomic technologies were to enable *multi-cancer* screening. Screening participants would be confronted with multiple and heterogeneous results, comprising cancer risks that might range from very low to very high risk. Moreover, in risk-stratified screening programmes, the results would lead to various sets of recommendations tailored to various risk groups. This means that during the informed consent process, prospective participants might need to be informed about various possible outcomes and subsequent follow-up trajectories (and corresponding implications thereof) before deciding to take part in screening. Providers of epigenomics-based screening programmes will need to participate in sustained efforts to inform and educate the public about the meaning of (epigenomic) cancer risk to ensure informed consent for screening, and, in doing so, take existing guidance into account. Hofmann has offered five guiding principles for information provision on mammographic screening, which might be helpful for epigenomics-based screening programmes: uncertainties should be acknowledged, information should be balanced, and the voluntariness of participation should be stressed.^37^ Also, stakeholders, including experts and prospective participants, should be involved in developing informational materials, and information should be presented in a layered fashion.^37^ Furthermore, providers should be careful with the terminology used in information provision and communication. It is essential to ensure that participants with high-risk results do not consider themselves cancer patients. Also in the context of breast cancer screening, Rainey et al^38^ suggested using the term ‘risk reduction’ in lieu of ‘cancer prevention’.

Second, this study shows that experts are concerned that epigenomic screening may increase the tendency within society to hold individuals responsible for their health, resulting in blaming the victim, stigmatisation and discrimination. Similar concerns were found in a quantitative study of attitudes regarding epigenetic screening among women in five European countries.^39^ Although the majority of women surveyed were interested in predictive epigenetic testing for female cancers for its potential to guide cancer prevention strategies and lifestyle adaptations and its perceived positive benefit-to-risk ratio, they were concerned about unnecessary worry, a reduced quality of life and pressure on women to adopt healthier lifestyles or take part in more cancer screening.^39^ In epigenetics, the question of personal responsibility for health is accorded new importance, as epigenetics may expose causal relationships between lifestyle or environmental factors and an individual’s health condition. Moreover, epigenetic risk is – in principle – reversible, and risk can be reduced through targeted preventive interventions. Using epigenetic technology to measure and monitor the risk-reducing effects of interventions over time, individuals might gain real-time insight into their disease risks and the effects of lifestyle or environmental interventions on these risks. If this knowledge and these technological possibilities become increasingly available to us, we might be held responsible if we fail to make use of them.^15^ This may not be fair, as people rarely willingly choose not to adopt healthier lifestyles, and generally find it difficult to change their habits; studies of the effects of (epi)genetic risk information on behaviour change show mixed results.^40,41^ Moreover, the precise causal role of individual lifestyle-related, genetic and environmental factors in the aetiology of disease may be complex and difficult to disentangle, and thus caution should be exercised in the normative translation of epigenetic research results.^42^

The phenomenon of over-responsibilisation may be aggravated by the present (or future) context in which potential new epigenomic-based screening programmes might be introduced, alongside a growing range of health-promoting screening tests, health checks and preventive measures, offered by the state, healthcare professionals or private parties. The current ‘omnipresence’ of health checks may have effects on individuals over and above the effects of discrete health checks or screening programmes, notably in violating their ‘privacy and peace of mind’.^43^ When people are repeatedly confronted with screening offers, they are repeatedly asked to make decisions whether or not to take up on these offers, which may constitute a burden. In society, some form of pressure may arise to participate in screening and/or to adopt a healthier lifestyle, and it may become more difficult for citizens to withdraw from or evade screening.^44^ Another concern related to responsibility, which was mentioned in our focus groups, is a (misplaced) focus on personal responsibility for health may direct attention away from important and modifiable structural and societal causative factors for ill health. These factors can and should be addressed by state or healthcare actors, not by individuals.^16,17,45^

Third, adequate protection and governance of epigenomic data is considered a precondition for any morally responsible epigenomics-based screening programme. Increasingly, pseudonymised epigenomic research data sets are made accessible to the public (ie, open access) or to other researchers (ie, controlled access) in online repositories, to allow for secondary use of data for research purposes, and therewith, to contribute to a better understanding of epigenomics and its effects on human health and disease. While this may lead to privacy concerns among researchers,^46^ mechanisms are being developed to adequately protect data and samples from research participants or biobank donors in research settings.^47,48^ It is a subject of debate whether and to what extent screening organisations may make data or samples available for secondary research purposes. It would be useful, in this context, to study the preferences of the public, particularly (prospective) screening participants, in relation to data re-use. It should be noted that, in practice, privacy concerns need not always arise. This would be highly dependent on the future design of the epigenomic technology. The screening test could be designed such that it entails no more than a targeted analysis of a limited set of DNA methylation markers, for instance, and that this set of markers reveals only information about (preliminary stages and/or risk of) cervical cancer, and is not connected to any environmental or lifestyle exposures. Also, screening tests could be devised such that there is only a very slim chance or no chance at all of detecting incidental findings related to risks for other conditions. If the epigenomics-based screening for cervical cancer were designed as described, it would be similar to current screening modalities, such as tests that detect infection with human papillomavirus. From an ethical point of views, there would be no difference, and epigenomics-specific privacy concerns would not arise.

Fourth and finally, there are concerns that for the (ethical) evaluation of epigenomics-based screening programmes, the criteria offered by Wilson and Jungner^49^ on behalf of the World Health Organisation (WHO) in the late 1960s, will no longer suffice. The WHO criteria have served as the foundations for national and international norms and regulations for screening programmes around the world. In 2008, the framework was expanded to accommodate genetic and genomic screening, and came to include additional criteria, including a well-defined targeted group, scientific evidence for the cost-effectiveness of the screening programme, respect for the autonomy of and informed choice by screening participants, privacy protection and equal access.^50^ The core tenet of this – internationally broadly supported – ethical framework is that screening is always associated with burdens and potential harms, and is therefore only justified when the benefits clearly outweigh the burdens and risks. In other words, screening must be proportional. However, epigenomics-based screening may not easily meet the criteria of the existing framework. How to apply the first criterion: ‘the condition sought should be an important health problem’? Should all diseases included in the test be (equally) important health problems, or should they be taken together? Must all cancer risk tests included in the screening offer be considered as having a ‘recognizable latent or early symptomatic stage’ (the fourth criterion)? And will the test be ‘acceptable to the population’ (the sixth criterion) if it implies that certain groups will be offered less frequent screening as a result of risk stratification? We have already seen that there are concerns that information provision and informed consent may be difficult for complex epigenomics-based screening programmes, involving risk stratification and multi-cancer screening (the 17th criterion of the expanded framework). Most importantly, when screening programmes have mostly individualised health recommendations and lifestyle advice to offer, the benefits of screening are not likely to outweigh the risks, as tailored lifestyle advice may not result in health gains. As one of our focus group respondents noted, there is no use in offering ‘personalised screening for general health recommendations’ (Focus group B, application 3); one might simply omit (potentially costly) screening and proceed to offering heath recommendations and other general preventive measures directly. Interestingly, the introduction of risk stratification approaches might lead population screening programmes to lose (part of) their programmatic character, when the target population is subdivided into smaller groups with shared risks, and participants are offered recommendations, preventive measures or adapted screening, tailored to their individual risks. Slowly, screening might begin to assume the character of individual (preventive) care. Also, it might become more difficult to monitor and ensure the quality (based on data) of screening programmes when these consist of multiple sub-programmes tailored to smaller groups with shared risks.^51^

This study has several limitations. First, our sample was small (n = 13) and Dutch only. We did recruit respondents from ethics and/or philosophy departments at universities from across the country. Also, we managed to include only 4 to 5 respondents per focus group discussion, whereas most focus group studies include 6 to 8 respondents. Additional focus groups – in other countries – might have elicited additional topics. Further focus group studies might be conducted internationally to broaden the scope of our work. Second, the level of knowledge of epigenetics and epigenomics varied among respondents. Our main inclusion criterion was participants’ expertise in the identification of the ethical implications of emerging (bio)medical technologies. Given the relative novelty of epigenomics as a field of research, there are very few researchers – in our country – that combine expertise in the ethics of medical innovations with specific technical expertise in epigenetics and epigenomics. Ideally, future empirical work might engage such experts. Third, as our second potential application of epigenomic technology in screening was *women-specific* multi-cancer screening, focus group discussions may have focussed disproportionally on responsibilisation and burdens for women.

## Conclusion

Although epigenomics technology might offer insight into potentially modifiable individual cancer risks, it remains to be seen whether it can be used – in combination with other omics technologies – for the prediction of cancer and the stratification of risk. If it can, the benefits of any epigenomics-based screening programmes should outweigh the burdens and potential harms. Further research should demonstrate whether epigenomic risk information will motivate individuals to adopt healthier lifestyles or avoid environmental exposures. To realise health benefit on a population level, citizens should not only be informed and educated about epigenomic risk, but also given the opportunity to reduce epigenomic risk and take accessible and effective preventive action, while being protected against ‘over-responsiblisation’ or societal pressure and stigmatisation. Given the particular sensitivity of epigenomic data, screening organisations may need to take privacy concerns into account in the design and development of the screening test, by ensuring, for instance, that it conveys information about cancer risk only, not about causes of cancer that may trigger blame discourses. Finally, the potential future application of risk stratification based on epigenomic risk information in screening programmes may have implications for the evaluation of screening programmes, and a rethinking of the existing ethical framework for screening might be required.
